# A note on “Implicit iterative process with errors”

**DOI:** 10.1186/s40064-016-1964-4

**Published:** 2016-03-15

**Authors:** Esra Yolacan

**Affiliations:** Department of Mathematics, Faculty of Science, Ataturk University, Erzurum, 25240 Turkey

**Keywords:** Total asymptotically nonexpansive mappings, Common fixed points, Implicit iterative process with errors, 47H09, 47H10, 46B20

## Abstract

In this paper, I introduce an implicit iterative process with mixed errors for two finite family of total asymptotically nonexpansive mappings in a uniformly convex Banach space and prove strong convergence theorems under some conditions. My results improved and extended many know results existing in the literature.

## Background

Let *E* is a normed space and *K* be a nonempty subset of *E*. I also assume that $\phi : \mathbb {R} ^{+}\rightarrow \mathbb {R} ^{+}$strictly increasing continuous function with $\phi (0)=0$. Let $T:K\rightarrow K$ be a mapping. A point $x\in K$ is called a fixed point of *T* if and only if $Tx=x$. I will denote by nonexpansive if $F\left( T\right)$ the set of fixed points of *T*, that is, $F(T):=\left\{ x\in K:Tx=x\right\}$. *T* is said to be *nonexpansive* if

$$\begin{aligned} \left\| Tx-Ty\right\| \le \left\| x-y\right\| \end{aligned}$$

for all $x, y\in K$. *T* is called *asymptotically nonexpansive* if for a sequence $\left\{ k_{n}\right\} \subset \left[ 1,\infty \right)$ with $k_{n}\rightarrow 1$,

$$\begin{aligned} \left\| T^{n}x-T^{n}y\right\| \le k_{n}\left\| x-y\right\| \end{aligned}$$

for all $x, y\in K$ and $n\ge 1$. *T* is said to be *totalasymptoticallynonexpansive* (see, e.g., Albert et al. 2006) if

$$\begin{aligned} \left\| T^{n}x-T^{n}y\right\| \le \left\| x-y\right\| +\mu _{n}\phi (\left\| x-y\right\| )+l_{n} \end{aligned}$$

for all $x, y\in K, n\ge 1$ where $\left\{ \mu _{n}\right\}$ and $\left\{ l_{n}\right\}$ nonnegative real sequences such that $\mu _{n}, l_{n}\rightarrow 0$ as $n\rightarrow \infty$. From the definition, I see that the class of total asymptotically nonexpansive mappings include the class of asymptotically nonexpansive mappings as a special case; see also Albert et al. (2006) for more details.

### **Proposition 1**

*LetKbe a nonempty subset of*$E,\left\{ S_{i}\right\} _{i=1}^{N},\left\{ T_{i}\right\} _{i=1}^{N}:K\rightarrow K$*be* 2*Ntotal asymptotically nonexpansive mappings. Then there exist nonnegative real sequences*$\left\{ \mu _{n}\right\}$*and*$\left\{ l_{n}\right\} , n\ge 1$*with*$\mu _{n}, l_{n}\rightarrow 0$*as*$n\rightarrow \infty$*and strictly increasing continuous function*$\phi : \mathbb {R} ^{+}\rightarrow \mathbb {R} ^{+}$*with*$\phi (0)=0$*such that for all*$x, y\in K,$

1 $$\begin{aligned} \left\| T_{i}^{n}x-T_{i}^{n}y\right\|\le \left\| x-y\right\| +\mu _{n}\phi \left( \left\| x-y\right\| \right) +l_{n},\quad n\ge 1, \end{aligned}$$

2 $$\begin{aligned} \left\| S_{i}^{n}x-S_{i}^{n}y\right\|\le  \left\| x-y\right\| +\mu _{n}\phi \left( \left\| x-y\right\| \right) +l_{n},\quad n\ge 1, \quad \hbox{for}\,i=1,2,\ldots ,N.\end{aligned}$$

### *Proof*

Since $T_{i}:K\rightarrow K$ is a total asymptotically nonexpansive mappings for $i=1,2,\ldots ,N,$ there exist nonnegative real sequences $\left\{ \mu _{in}\right\}$, $\left\{ l_{in}\right\}$, $n\ge 1$ with $\mu _{in}, l_{in}\rightarrow 0$ as $n\rightarrow \infty$ and strictly increasing continuous function $\phi _{i}: \mathbb {R} ^{+}\rightarrow \mathbb {R} ^{+}$ with $\phi _{i}\left( 0\right) =0$ such that for all $x, y\in K,$

$$\begin{aligned} \left\| T_{i}^{n}x-T_{i}^{n}y\right\| \le \left\| x-y\right\| +\mu _{in}\phi _{i}\left( \left\| x-y\right\| \right) +l_{in},\quad n\ge 1. \end{aligned}$$

Setting

$$\begin{aligned} \mu _{n}&=  \max \left\{ \mu _{1n},\mu _{2n},\ldots ,\mu _{Nn}\right\} , \quad l_{n}=\max \left\{ l_{1n},l_{2n},\ldots ,l_{Nn}\right\} ,\\ \phi \left( a\right)&=  \max \left\{ \phi _{1}\left( a\right) ,\phi _{2}\left( a\right) ,\ldots ,\phi _{N}\left( a\right) \right\} \text { for }a\ge 0, \end{aligned}$$

then I get that there exist nonnegative real sequences $\left\{ \mu _{n}\right\}$ and $\left\{ l_{n}\right\} , n\ge 1$ with $\mu _{n}, l_{n}\rightarrow 0$ as $n\rightarrow \infty$ and strictly increasing continuous function $\phi : \mathbb {R} ^{+}\rightarrow \mathbb {R} ^{+}$ with $\phi \left( 0\right) =0$ such that

$$\begin{aligned} \left\| T_{i}^{n}x-T_{i}^{n}y\right\|&\le \left\| x-y\right\| +\mu _{in}\phi _{i}\left( \left\| x-y\right\| \right) +l_{in} \\ &\le {} \left\| x-y\right\| +\mu _{n}\phi \left( \left\| x-y\right\| \right) +l_{n},\quad n\ge 1, \end{aligned}$$

for all $x,y\in K,$ and each $i=1,2,\ldots ,N.$

In a way similar to the above, I can also prove (2).□

Recently, fixed point problems based on implicit iterative processes have been considered by many authors, (see, for example, Chang et al. 2006; Cianciaruso et al. 2010; Sun 2003; Gu 2006; Qin et al. 2008; Xu and Ori 2001). In Hao (2010) established weak and strong convergence theorems of the implicit iteration process for a finite family of uniformly Lipschitz total asymptotically nonexpansive mappings in a real Hilbert space. In Hao et al. (2012) studied weak and strong convergence theorems for common fixed points of two finite family of asymptotically nonexpansive mappings in a uniformly convex Banach space.

Note the convergence problems of an implicit (an explicit) iterative process to a common fixed point, for total asymptotically nonexpansive (or asymptotically nonexpansive) in Banach space have been obtained by a number of authors (see more details, Mukhamedov and Saburov 2010a, b, 2011, 2012a, b).

Inspired and motivated by this facts, I introduce an implicit iterative process with mixed errors for two finite family of total asymptotically nonexpansive mappings in a uniformly convex Banach space. The results of this paper can be viewed as an improvement and extension of the corresponding results of Chang et al. (2006), Cianciaruso et al. (2010), Sun (2003), Hao et al. (2012), Hao (2010) and others.

### **Definition 1**

Let $\left\{ S_{i}\right\} _{i=1}^{N}$, $\left\{ T_{i}\right\} _{i=1}^{N}:E\rightarrow E$ be 2*N* total asymptotically nonexpansive mappings. Define the sequence $\left\{ x_{n}\right\}$ as follows: $x_{0}\in K,$ and

3 $$\begin{aligned} x_{n} &= \left( 1-\alpha _{n}-\gamma _{n}\right) x_{n-1}+\alpha _{n}T_{i\left( n\right) }^{k\left( n\right) }y_{n}+\gamma _{n}u_{n}, \nonumber \\ y_{n} & =\left( 1-\beta _{n}-\delta _{n}\right) x_{n}+\beta _{n}S_{i\left( n\right) }^{k\left( n\right) }x_{n}+\delta _{n}v_{n}, n\ge 1 \end{aligned}$$

where $n=\left( k\left( n\right) -1\right) N+i\left( n\right)$, $i\left( n\right) \in 1,2,\ldots ,N$, $\left\{ \alpha _{n}\right\} ,\left\{ \beta _{n}\right\} ,\left\{ \gamma _{n}\right\}$ and $\left\{ \delta _{n}\right\}$ are four real sequences in $\left[ 0,1\right]$ satisfying $\alpha _{n}+\gamma _{n}\le 1$ and $\beta _{n}+\delta _{n}\le 1$ for all $n\ge 1,\left\{ u_{n}\right\}$ and $\left\{ v_{n}\right\}$ are two bounded sequences.

The purpose of this paper is to study the strong convergence of implicit iterative process with mixed errors for two finite family of total asymptotically nonexpansive mappings in Banach spaces.

## Preliminaries

Let *E* be a Banach space with dimension $E\ge 2$. The modulus of *E* is the function $\delta _{E}:\left( 0,2\right] \rightarrow \left[ 0,1\right]$ defined by

$$\begin{aligned} \delta _{E}\left( \varepsilon \right) =\inf \left\{ 1-\left\| \frac{1}{2} (x+y)\right\| :\mathrm {\;}\left\| x\right\| =\left\| y\right\| =1,\mathrm {\;}\varepsilon =\left\| x-y\right\| \right\} . \end{aligned}$$

A Banach space *E* is uniformly convex if and only if $\delta _{E}\left( \varepsilon \right) >0$ for all $\varepsilon \in \left( 0,2\right]$.

Recall that a mapping $T:K\rightarrow K$ is $semi-compact$ ($or hemi-compact$) if any sequence $\left\{ x_{n}\right\}$ in *K* such that $\left\{ x_{n}-Tx_{n}\right\} \rightarrow 0$ as $n\rightarrow \infty$ has a convergent subsequence.

### **Lemma 1**

(Tan and Xu 1993) *Let*$$\left\{ a_{n}\right\}, $$$$\left\{ b_{n}\right\} $$*and*$$\left\{ \delta _{n}\right\} $$*be sequences of nonnegative real numbers satisfying the inequality*

$$\begin{aligned} a_{n+1}\le \left( 1+\delta _{n}\right) a_{n}+b_{n},\quad n\ge 1, \end{aligned}$$

*if*$\sum _{n=1}^{\infty }b_{n}<\infty$*and*$\sum _{n=1}^{\infty }\delta _{n}<\infty$, *then*

(i) $$\lim \limits _{n\rightarrow \infty }a_{n}$$*exists*;
(ii) *In particular, if*$\left\{ a_{n}\right\}$*has a subsequence*$\left\{ a_{n_{k}}\right\}$*converging to* 0, then $\lim \limits _{n\rightarrow \infty }a_{n}=0.$

### **Lemma 2**

(Schu 1991) *LetEbe a uniformly convex Banach space*, $\left\{ t_{n}\right\} _{n\ge 1}\subseteq \left[ b,c\right] \subset \left( 0,1\right)$, $\left\{ x_{n}\right\} _{n\ge 1}$*and*$\left\{ y_{n}\right\} _{n\ge 1}$*be sequences inE*. *If*$\lim \sup _{n\rightarrow \infty }\left\| x_{n}\right\| \le a$, $\lim \sup _{n\rightarrow \infty }\left\| y_{n}\right\| \le a$*and*$\lim _{n\rightarrow \infty }\left\| t_{n}x_{n}+\left( 1-t_{n}\right) y_{n}\right\| =a$*for some constant*$a\ge 0,$*then*$\lim _{n\rightarrow \infty }\left\| x_{n}-y_{n}\right\| =0.$

## Main results

### **Lemma 3**

*LetEbe a real Banach space, letKbe a nonempty,closed and convex subset ofEand*$\left\{ S_{i}\right\} _{i=1}^{N}$, $\left\{ T_{i}\right\} _{i=1}^{N}:K\rightarrow K$*be* 2*Ntotal asymptotically nonexpansive mappings*$\left\{ \mu _{n}\right\}$, $\left\{ l_{n}\right\}$*defined by* (1) and (2) *such that*

4 $$\begin{aligned} \sum \limits _{n=1}^{\infty }\mu _{n}<\infty ,\quad \sum \limits _{n=1}^{\infty }l_{n}<\infty \end{aligned}$$

*and*$${\mathcal {F}}:\bigcap \nolimits _{i=1}^{N}F\left( T_{\dot{I}}\right) \cap F\left( S_{\dot{I}}\right) \ne \varnothing $$. *Assume that there existM*, $$ M^{*}>0$$*such that*$$\phi (\lambda )\le M^{*}\lambda $$ for all $$ \lambda \ge M$$, $$i\in \left\{ i=1,2,\ldots ,N\right\} .$$*Let*$$\left\{ u_{n}\right\} $$*and*$$\left\{ v_{n}\right\} $$*are two bounded sequences inK*. *Let*$$\left\{ \alpha _{n}\right\} ,\left\{ \beta _{n}\right\} ,\left\{ \gamma _{n}\right\} $$*and*$$\left\{ \delta _{n}\right\} $$*be four real sequences in*$$\left[ 0,1\right] $$*satisfying the following conditions:*

(i) $\alpha _{n}+\gamma _{n}\le 1$*and*$\beta _{n}+\delta _{n}\le 1$*for all*$n\ge 1;$
(ii) $$\lim \sup _{n\rightarrow \infty }\alpha _{n}<1;$$
(iii) $$\sum \nolimits _{n=1}^{\infty }\gamma _{n}<\infty $$, $$\sum \nolimits _{n=1}^{ \infty }\delta _{n}<\infty .$$
  *Starting from an arbitrary*$x_{0}\in K$, *define the sequence*$\left\{ x_{n}\right\}$*by recursion* (3). *Then*, $\lim _{n\rightarrow \infty }\left\| x_{n}-p\right\|$*exists for all*$p\in {\mathcal {F}}.$

### *Proof*

Let $p\in {\mathcal {F}}$. Since $\left\{ u_{n}\right\}$ and $\left\{ v_{n}\right\}$ are two bounded sequences in *K*, I have

$$\begin{aligned} K=\max \left\{ \sup _{n\ge 1}\left\| u_{n}-p\right\| ,\sup _{n\ge 1}\left\| v_{n}-p\right\| \right\} . \end{aligned}$$

Since $S_{1},S_{2},\ldots ,S_{N}$ are total asymptotically nonexpansive mappings, it follows from (2) that

5 $$\begin{aligned} \left\| y_{n}-p\right\| & = \left\| \left( 1-\beta _{n}-\delta _{n}\right) \left( x_{n}-p\right) +\beta _{n}\left( S_{i\left( n\right) }^{k\left( n\right) }x_{n}-p\right) +\delta _{n}\left( v_{n}-p\right) \right\| \nonumber \\ &\le \left( 1-\beta _{n}-\delta _{n}\right) \left\| x_{n}-p\right\| +\beta _{n}\left\| S_{i\left( n\right) }^{k\left( n\right) }x_{n}-p\right\| +\delta _{n}\left\| v_{n}-p\right\| \\ &\le \left( 1-\beta _{n}-\delta _{n}\right) \left\| x_{n}-p\right\| +\beta _{n}\left[ \left\| x_{n}-p\right\| +\mu _{n}\phi \left( \left\| x_{n}-p\right\| \right) +l_{n}\right]   +\delta _{n}K \nonumber \\ &\le \left\| x_{n}-p\right\| +\beta _{n}\mu _{n}\phi \left( \left\| x_{n}-p\right\| \right) +\beta _{n}l_{n}+\varphi _{\left( 1\right) }^{n}, \end{aligned}$$

where $\varphi _{\left( 1\right) }^{n}=\delta _{n}K$. Since $\sum \nolimits _{n=1}^{\infty }\delta _{n}<\infty$, I can see that $\sum \nolimits _{n=1}^{\infty }\varphi _{\left( 1\right) }^{n}<\infty$. Note that $\phi$ is an increasing function, it follows that $\phi \left( \lambda \right) \le \phi \left( M\right)$ whenever $\lambda \le M$ and ( by hypothesis) $\phi \left( \lambda \right) \le M^{*}\lambda$ if $\lambda \ge M$. In either case, I have

6 $$\begin{aligned} \phi \left( \lambda \right) \le \phi \left( M\right) +M^{*}\lambda , \end{aligned}$$

for some $M,M^{*}>0$. Thus, from (5) and (6), I have

7 $$\begin{aligned} \left\| y_{n}-p\right\|&\le \left\| x_{n}-p\right\| +\beta _{n}\mu _{n}\left[ \phi \left( M\right) +M^{*}\left\| x_{n}-p\right\| \right]+\beta _{n}l_{n}+\varphi _{\left( 1\right) }^{n} \nonumber \\ &\le \left( 1+M^{*}\mu _{n}\right) \left\| x_{n}-p\right\| +R_{1}\left( \mu _{n}+l_{n}\right) +\varphi _{\left( 1\right) }^{n}, \end{aligned}$$

for some constant $R_{1}>0$. It follows from (6) and (7)

8 $$\begin{aligned} \left\| x_{n}-p\right\| &= \left\| \left( 1-\alpha _{n}-\gamma _{n}\right) \left( x_{n-1}-p\right) +\alpha _{n}\left( T_{i\left( n\right) }^{k\left( n\right) }y_{n}-p\right) +\gamma _{n}\left( u_{n}-p\right) \right\| \nonumber \\ &\le \left( 1-\alpha _{n}-\gamma _{n}\right) \left\| x_{n-1}-p\right\| +\alpha _{n}\left\| T_{i\left( n\right) }^{k\left( n\right) }y_{n}-p\right\| +\gamma _{n}\left\| u_{n}-p\right\| \nonumber \\ &\le \left( 1-\alpha _{n}-\gamma _{n}\right) \left\| x_{n-1}-p\right\| +\alpha _{n}\left[ \left\| y_{n}-p\right\| +\mu _{n}\phi \left( \left\| y_{n}-p\right\| \right) +l_{n}\right] \nonumber \\ &\quad +\gamma _{n}\left\| u_{n}-p\right\| \nonumber \\ &\le \left( 1-\alpha _{n}-\gamma _{n}\right) \left\| x_{n-1}-p\right\| \nonumber \\ &\quad +\alpha _{n}\left[ \left( 1+M^{*}\mu _{n}\right) \left\| x_{n}-p\right\| +R_{1}\left( \mu _{n}+l_{n}\right) +\varphi _{\left( 1\right) }^{n}\right] \nonumber \\ &\quad +\alpha _{n}\mu _{n}\left[ \phi \left( M\right) +M^{*}\left\| y_{n}-p\right\| \right] +\alpha _{n}l_{n}+\gamma _{n}K \nonumber \\ &\le \left( 1-\alpha _{n}-\gamma _{n}\right) \left\| x_{n-1}-p\right\| +\alpha _{n}\left\| x_{n}-p\right\| \nonumber \\ &\quad +\alpha _{n}M^{*}\left( 2+M^{*}\mu _{n}\right) \mu _{n}\left\| x_{n}-p\right\| \nonumber \\ &\quad +\alpha _{n}R_{1}\left( \mu _{n}+l_{n}\right) +\alpha _{n}\varphi _{\left( 1\right) }^{n}+\alpha _{n}\mu _{n}\phi \left( M\right) +\alpha _{n}l_{n}+\gamma _{n}K \nonumber \\  &\quad +\alpha _{n}\mu _{n}M^{*}R_{1}\left( \mu _{n}+l_{n}\right) +\alpha _{n}\mu _{n}M^{*}\varphi _{\left( 1\right) }^{n} \nonumber \\ &\le \left( 1-\alpha _{n}\right) \left\| x_{n-1}-p\right\| +\alpha _{n}\left( 1+M_{2}\mu _{n}\right) \left\| x_{n}-p\right\| \nonumber \\ &R_{2}\left( \mu _{n}+l_{n}\right) +\alpha _{n}\varphi _{\left( 1\right) }^{n}+\gamma _{n}K+\alpha _{n}\mu _{n}M^{*}\varphi _{\left( 1\right) }^{n}, \end{aligned}$$

for some constants $M_{2},R_{2}>0$. I note that

9 $$\begin{aligned} \left\| x_{n}-p\right\| &\le \left( 1-\alpha _{n}\right) \left\| x_{n-1}-p\right\| +\alpha _{n}\left( 1+M_{2}\mu _{n}\right) \left\| x_{n}-p\right\| \nonumber \\ &\quad +R_{2}\left( \mu _{n}+l_{n}\right) +\varphi _{\left( 2\right) }^{n}, \end{aligned}$$

where $\varphi _{\left( 2\right) }^{n}=\alpha _{n}\varphi _{\left( 1\right) }^{n}+\gamma _{n}K+\alpha _{n}\mu _{n}M^{*}\varphi _{\left( 1\right) }^{n}$. Since $\sum \nolimits _{n=1}^{\infty }\gamma _{n}<\infty$, $\sum \nolimits _{n=1}^{\infty }\varphi _{\left( 1\right) }^{n}<\infty$ , I can see that $\sum \nolimits _{n=1}^{\infty }\varphi _{\left( 2\right) }^{n}<\infty$. This implies that

10 $$\begin{aligned} \left\| x_{n}-p\right\| &\le \frac{1-\alpha _{n}}{1-\alpha _{n}\left( 1+M_{2}\mu _{n}\right) }\left\| x_{n-1}-p\right\| +\frac{ R_{2}\left( \mu _{n}+l_{n}\right) +\varphi _{\left( 2\right) }^{n}}{1-\alpha _{n}\left( 1+M_{2}\mu _{n}\right) } \nonumber \\ &\le \left( 1+\frac{\alpha _{n}M_{2}\mu _{n}}{1-\alpha _{n}\left( 1+M_{2}\mu _{n}\right) }\right) \left\| x_{n-1}-p\right\| +\frac{ R_{2}\left( \mu _{n}+l_{n}\right) +\varphi _{\left( 2\right) }^{n}}{1-\alpha _{n}\left( 1+M_{2}\mu _{n}\right) }. \end{aligned}$$

By hypothesis (ii), it follows that there exists $\lambda <1$, such that $\alpha _{n}\le \lambda$ for big *n*. It follows that

$$\begin{aligned} \frac{\alpha _{n}M_{2}}{1-\alpha _{n}\left( 1+M_{2}\mu _{n}\right) }\le \frac{\lambda M_{2}}{1-\lambda \left( 1+M_{2}\mu _{n}\right) }. \end{aligned}$$

From $\lim _{n\rightarrow \infty }\mu _{n}=0$, it derives that $\lim _{n\rightarrow \infty }\frac{\lambda M_{2}}{1-\lambda \left( 1+M_{2}\mu _{n}\right) }=\frac{\lambda M_{2}}{1-\lambda }$. Then there exists a real constant $L_{1}>0$ such that

$$\begin{aligned} \frac{\lambda M_{2}}{1-\lambda \left( 1+M_{2}\mu _{n}\right) }\le L_{1},\quad  \forall n\ge 1. \end{aligned}$$

It follows from the hypothesis that $\sum _{n\ge 1}\frac{\alpha _{n}M_{2}}{ 1-\alpha _{n}\left( 1+M_{2}\mu _{n}\right) }<\infty$. Similarly, I can prove that

11 $$\begin{aligned} \sum _{n\ge 1}\frac{R_{2}\left( \mu _{n}+l_{n}\right) +\varphi _{\left( 2\right) }^{n}}{1-\alpha _{n}\left( 1+M_{2}\mu _{n}\right) }<\infty . \end{aligned}$$

Besides, I can write

$$\begin{aligned} \left\| x_{n}-p\right\| \le \left( 1+L_{1}\mu _{n}\right) \left\| x_{n-1}-p\right\| +\frac{R_{2}\left( \mu _{n}+l_{n}\right) +\varphi _{\left( 2\right) }^{n}}{1-\alpha _{n}\left( 1+M_{2}\mu _{n}\right) }, \end{aligned}$$

where a real constant $L_{1}>0$. Thus, I obtain from it that

12 $$\begin{aligned} \left\| x_{n}-p\right\| \le \left( 1+\delta _{n}\right) \left\| x_{n-1}-p\right\| +b_{n},\quad \forall n\ge n_{0}, \end{aligned}$$

where $\delta _{n}=L_{1}\mu _{n}$ and $b_{n}=\frac{R_{2}\left( \mu _{n}+l_{n}\right) +\varphi _{\left( 2\right) }^{n}}{1-\alpha _{n}\left( 1+M_{2}\mu _{n}\right) }$ and using the condition (iii) and (11), it is easy to see that $\sum _{n=1}^{\infty }\delta _{n}<\infty$ and $\sum _{n=1}^{\infty }b_{n}<\infty$. In view of Lemma 1, I find that $\lim _{n\rightarrow \infty }\left\| x_{n}-p\right\|$ exist for all $p\in {\mathcal {F}}$. $\square$

### **Theorem 1**

*LetEbe a real uniformly convex Banach space, letKbe a nonempty,closed and convex subset ofEand*$\left\{ S_{i}\right\} _{i=1}^{N}$, $\left\{ T_{i}\right\} _{i=1}^{N}:K\rightarrow K$ be 2*Ntotal asymptotically nonexpansive mappings*$\left\{ \mu _{n}\right\}$, $\left\{ l_{n}\right\}$*defined by* (1) *and* (2) *such that*

13 $$\begin{aligned} \sum \limits _{n=1}^{\infty }\mu _{n}<\infty ,\quad \sum \limits _{n=1}^{\infty }l_{n}<\infty \end{aligned}$$

*and*$${\mathcal {F}}:\bigcap \nolimits _{i=1}^{N}F\left( T_{\dot{I}}\right) \cap F\left( S_{\dot{I}}\right) \ne \varnothing $$. *Assume that there existM*, $$ M^{*}>0$$*such that*$$\phi (\lambda )\le M^{*}\lambda $$*for all*$$ \lambda \ge M$$, $$i\in \left\{ i=1,2,\ldots ,N\right\} .$$*Let*$$\left\{ u_{n}\right\} $$*and*$$\left\{ v_{n}\right\} $$*are two bounded sequences inK*. *Let*$$\left\{ \alpha _{n}\right\} ,\left\{ \beta _{n}\right\} ,\left\{ \gamma _{n}\right\} $$*and*$$\left\{ \delta _{n}\right\} $$*be four real sequences in*$$\left[ 0,1\right] $$*satisfying the following conditions:*

(i) $\alpha _{n}+\gamma _{n}\le 1$*and*$\beta _{n}+\delta _{n}\le 1$*for all*$n\ge 1;$
(ii) $$\lim \sup _{n\rightarrow \infty }\alpha _{n}<1;$$
(iii) $$\sum \nolimits _{n=1}^{\infty }\gamma _{n}<\infty $$, $$\sum \nolimits _{n=1}^{ \infty }\delta _{n}<\infty .$$

*Then the implicit iterative sequence*$\left\{ x_{n}\right\}$*by* (3) *converges strongly to a common fixed point in*${\mathcal {F}}$*if and only if*$\lim \inf _{n\rightarrow \infty }d\left( x_{n},{\mathcal {F}}\right) =0$, *where*$d\left( x,{\mathcal {F}}\right)$*denotes the distance ofxto set*${\mathcal {F}}$, *i.e*., $d\left( x,{\mathcal {F}}\right) =\inf _{y\in {\mathcal {F}}}d\left( x,y\right)$.

### *Proof*

It suffices to show that $\lim \inf _{n\rightarrow \infty }d\left( x_{n},{\mathcal {F}}\right) =0$ implies that $\left\{ x_{n}\right\}$ converges to a common fixed point of ${\mathcal {F}}.$

Necessity. Since (12) holds for all $p\in {\mathcal {F}}$, I obtain from it that

$$\begin{aligned} d\left( x_{n},{\mathcal {F}}\right) \le \left( 1+\delta _{n}\right) d\left( x_{n-1},{\mathcal {F}}\right) +b_{n},\quad \forall n\ge n_{0}, \end{aligned}$$

Lemma 1 that $\lim _{n\rightarrow \infty }d\left( x_{n},{\mathcal {F}}\right)$ exists and so $\lim _{n\rightarrow \infty }d\left( x_{n},{\mathcal {F}}\right) =0$.

Sufficiency. Now, I show that $\left\{ x_{n}\right\}$ is a Cauchy sequence in *E*. For any positive integers $m,n>n\ge n_{0}$, from $1+t\le e^{t}$ for all $t>0$ and (12), I have

$$\begin{aligned} \left\| x_{m}-p\right\| &\le \left( 1+\delta _{m}\right) \left\| x_{m-1}-p\right\| +b_{m} \\ & \le e^{\delta _{m}}\left\| x_{m-1}-p\right\| +b_{m} \\ &\le e^{\delta _{m}}\left( e^{\delta _{m-1}}\left\| x_{m-2}-p\right\| +b_{m-1}\right) +b_{m} \\ &\quad \vdots \\ &\le e^{\sum _{i=n+1}^{m}\delta _{i}}\left\| x_{n}-p\right\| +\sum _{k=n+1}^{m-1}b_{k}e^{\sum _{i=k+1}^{m}\delta _{i}}+b_{m} \\ &\le Q\left\| x_{n}-p\right\| +Q\sum _{k=n+1}^{\infty }b_{k}+b_{m}, \end{aligned}$$

where $Q=e^{\sum _{n=1}^{\infty }\delta _{n}}$. Thus for any $p\in {\mathcal {F}}$, I have

$$\begin{aligned} \left\| x_{n}-x_{m}\right\| &\le \left\| x_{n}-p\right\| +\left\| x_{m}-p\right\| \\ &\le \left( 1+Q\right) \left\| x_{n}-p\right\| +Q\sum _{k=n+1}^{\infty }b_{k}+b_{m}. \end{aligned}$$

Taking the infimum over all $p\in {\mathcal {F}}$, I obtain that

$$\begin{aligned} \left\| x_{n}-x_{m}\right\| \le \left( 1+Q\right) d\left( x_{n},{\mathcal {F}}\right) +Q\sum _{k=n+1}^{\infty }b_{k}+b_{m}. \end{aligned}$$

It follows from $\sum \nolimits _{n=1}^{\infty }b_{n}<\infty$ and $\lim _{n\rightarrow \infty }d\left( x_{n},{\mathcal {F}}\right) =0$ that $\left\{ x_{n}\right\}$ is a Cauchy sequence. Since *K* is a closed subset of *E* and so it is complete. Hence, there exists a $p\in K$ such that $x_{n}\rightarrow p$ as $n\rightarrow \infty$.

Finally, I have to prove that $p\in {\mathcal {F}}$. By contradiction, i assume that *p* is not in ${\mathcal {F}}:\bigcap \nolimits _{i=1}^{N}F\left( T_{ \dot{I}}\right) \cap F\left( S_{\dot{I}}\right) \ne \varnothing$. Since ${\mathcal {F}}$ is a closed set, $d\left( p,{\mathcal {F}}\right) >0$. Thus for all $p\in {\mathcal {F}}$, I have that

14 $$\begin{aligned} \left\| p-p_{1}\right\| \le \left\| p-x_{n}\right\| +\left\| x_{n}-p_{1}\right\| . \end{aligned}$$

This implies that

15 $$\begin{aligned} d\left( p,{\mathcal {F}}\right) \le \left\| p-x_{n}\right\| +d\left( x_{n},{\mathcal {F}}\right) . \end{aligned}$$

From (14) and (15) $\left( n\rightarrow \infty \right)$, I have that $d\left( p,{\mathcal {F}}\right) \le 0$. This is a contradiction. Thus, $p\in {\mathcal {F}}$. This completes the proof. $\square$

### **Lemma 4**

*LetEbe a uniformly convex Banach space, letKbe a nonempty,closed and convex subset ofEand*$\left\{ S_{i}\right\} _{i=1}^{N}$, $\left\{ T_{i}\right\} _{i=1}^{N}:K\rightarrow K$*be* 2*Nuniformly*$L_{i}-Lipschitz$*total asymptotically nonexpansive mappings*$\left\{ \mu _{n}\right\}$, $\left\{ l_{n}\right\}$*defined by* (1) and (2) *such that*

16 $$\begin{aligned} \sum \limits _{n=1}^{\infty }\mu _{n}<\infty ,\quad \sum \limits _{n=1}^{\infty }l_{n}<\infty \end{aligned}$$

*and*$${\mathcal {F}}:\bigcap \nolimits _{i=1}^{N}F\left( T_{\dot{I}}\right) \cap F\left( S_{\dot{I}}\right) \ne \varnothing $$. *Assume that there existM*, $$ M^{*}>0$$*such that*$$\phi (\lambda )\le M^{*}\lambda $$*for all*$$ \lambda \ge M$$, $$i\in \left\{ i=1,2,\ldots ,N\right\} .$$*Let*$$\left\{ u_{n}\right\} $$*and*$$\left\{ v_{n}\right\} $$*are two bounded sequences inK*. *Let*$$\left\{ \alpha _{n}\right\} ,\left\{ \beta _{n}\right\} ,\left\{ \gamma _{n}\right\} $$*and*$$\left\{ \delta _{n}\right\} $$*be four real sequences in*$$\left[ \frac{L-1}{L},a\right] $$, *where*$$L=\max _{1\le i\le N}\left\{ L_{i}\right\} >1$$*andais some constant in*$$\left( 0,1\right) $$*satisfying the following conditions:*

(i) $\alpha _{n}+\gamma _{n}\le 1$*and*$\beta _{n}+\delta _{n}\le 1$*for all*$n\ge 1;$
(ii) $$\lim \sup _{n\rightarrow \infty }\alpha _{n}<1;$$
(iii) $$\sum \nolimits _{n=1}^{\infty }\gamma _{n}<\infty $$, $$\sum \nolimits _{n=1}^{ \infty }\delta _{n}<\infty .$$

*Let the sequence*$\left\{ x_{n}\right\}$*and*$\left\{ y_{n}\right\}$*be defined by* (3). *Then*

$$\begin{aligned} \lim _{n\rightarrow \infty }\left\| x_{n}-T_{l}x_{n}\right\| &= 0, \\ \lim _{n\rightarrow \infty }\left\| x_{n}-S_{l}x_{n}\right\| &= 0, \\ \lim _{n\rightarrow \infty }\left\| S_{l}x_{n}-T_{l}x_{n}\right\| &= 0, \quad \forall l=1,2,\ldots ,N. \end{aligned}$$

### *Proof*

For all $p\in {\mathcal {F}}$, it follows from Lemma 3 that $\lim _{n\rightarrow \infty }\left\| x_{n}-p\right\|$ exists. Let $\lim _{n\rightarrow \infty }\left\| x_{n}-p\right\| =r$ for some $r\ge 0$. It follows from (7) that

17 $$\begin{aligned} \left\| y_{n}-p\right\| \le \left( 1+M^{*}\mu _{n}\right) \left\| x_{n}-p\right\| +R_{1}\left( \mu _{n}+l_{n}\right) +\varphi _{\left( 1\right) }^{n}, \end{aligned}$$

where $\sum \nolimits _{n=1}^{\infty }\mu _{n}<\infty ,\sum \nolimits _{n=1}^{\infty }l_{n}<\infty$ and $\sum \nolimits _{n=1}^{\infty }\varphi _{\left( 1\right) }^{n}<\infty$. Taking $\lim \sup _{n\rightarrow \infty }$ in both sides, I obtain

18 $$\begin{aligned} \lim \sup _{n\rightarrow \infty }\left\| y_{n}-p\right\|& \le  \lim \sup _{n\rightarrow \infty }\left[ \begin{array}{c} \left( 1+M^{*}\mu _{n}\right) \left\| x_{n}-p\right\| \\ +R_{1}\left( \mu _{n}+l_{n}\right) +\varphi _{\left( 1\right) }^{n} \end{array} \right] \nonumber \\ & \le r, \end{aligned}$$

and by (16) and (18)

19 $$\begin{aligned} \lim \sup _{n\rightarrow \infty }\left\| T_{i\left( n\right) }^{k\left( n\right) }y_{n}-p\right\|&\le \lim \sup _{n\rightarrow \infty }\left[ \begin{array}{c} \left\| y_{n}-p\right\| \\ +\mu _{n}\phi \left( \left\| y_{n}-p\right\| \right) +l_{n} \end{array} \right] \nonumber \\ &\le r. \end{aligned}$$

Notice that

20 $$\begin{aligned} \left\| T_{i\left( n\right) }^{k\left( n\right) }y_{n}-p+\gamma _{n}\left( u_{n}-x_{n-1}\right) \right\| \le \left\| T_{i\left( n\right) }^{k\left( n\right) }y_{n}-p\right\| +\gamma _{n}\left\| u_{n}-x_{n-1}\right\| . \end{aligned}$$

It follows that

21 $$\begin{aligned} \lim \sup _{n\rightarrow \infty }\left\| T_{i\left( n\right) }^{k\left( n\right) }y_{n}-p+\gamma _{n}\left( u_{n}-x_{n-1}\right) \right\| \le r, \end{aligned}$$

and

22 $$\begin{aligned} \left\| x_{n-1}-p+\gamma _{n}\left( u_{n}-x_{n-1}\right) \right\| \le \left\| x_{n-1}-p\right\| +\gamma _{n}\left\| u_{n}-x_{n-1}\right\| . \end{aligned}$$

These imply that

23 $$\begin{aligned} \lim \sup _{n\rightarrow \infty }\left\| x_{n-1}-p+\gamma _{n}\left( u_{n}-x_{n-1}\right) \right\| \le r, \end{aligned}$$

and I have that

24 $$\begin{aligned} x_{n}-p&= \alpha _{n}\left( T_{i\left( n\right) }^{k\left( n\right) }y_{n}-p+\gamma _{n}\left( u_{n}-x_{n-1}\right) \right) \nonumber \\ &+\left( 1-\alpha _{n}\right) \left( x_{n-1}-p+\gamma _{n}\left( u_{n}-x_{n-1}\right) \right) . \end{aligned}$$

Hence,

25 $$\begin{aligned} r &= \lim _{n\rightarrow \infty }\left\| x_{n}-p\right\| \nonumber \\ &= \lim _{n\rightarrow \infty }\left\| \begin{array}{c} \alpha _{n}\left( T_{i\left( n\right) }^{k\left( n\right) }y_{n}-p+\gamma _{n}\left( u_{n}-x_{n-1}\right) \right) \\ +\left( 1-\alpha _{n}\right) \left( x_{n-1}-p+\gamma _{n}\left( u_{n}-x_{n-1}\right) \right) \end{array} \right\| . \end{aligned}$$

Using (19), (23), (25) and Lemma 2, I find

26 $$\begin{aligned} \lim _{n\rightarrow \infty }\left\| T_{i\left( n\right) }^{k\left( n\right) }y_{n}-x_{n-1}\right\| =0. \end{aligned}$$

Notice that

$$\begin{aligned} \left\| x_{n}-T_{i\left( n\right) }^{k\left( n\right) }y_{n}\right\|&\le  \left\| x_{n}-x_{n-1}\right\| +\left\| x_{n-1}-T_{i\left( n\right) }^{k\left( n\right) }y_{n}\right\| \\ & \le \left( 1+\alpha _{n}\right) \left\| x_{n-1}-T_{i\left( n\right) }^{k\left( n\right) }y_{n}\right\| +\gamma _{n}\left\| u_{n}-x_{n-1}\right\| . \end{aligned}$$

It follows from (26) that

27 $$\begin{aligned} \lim _{n\rightarrow \infty }\left\| x_{n}-T_{i\left( n\right) }^{k\left( n\right) }y_{n}\right\| =0. \end{aligned}$$

Notice that

$$\begin{aligned} \left\| x_{n}-p\right\|&\le \left\| x_{n}-T_{i\left( n\right) }^{k\left( n\right) }y_{n}\right\| +\left\| T_{i\left( n\right) }^{k\left( n\right) }y_{n}-p\right\| \\ &\le \left\| x_{n}-T_{i\left( n\right) }^{k\left( n\right) }y_{n}\right\| \\ &\quad+\left\| y_{n}-p\right\| +\mu _{n}\phi \left( \left\| y_{n}-p\right\| \right) +l_{n}. \end{aligned}$$

By using (16) and (27), I obtain

$$\begin{aligned} r=\lim _{n\rightarrow \infty }\left\| x_{n}-p\right\| \le \lim \inf _{n\rightarrow \infty }\left\| y_{n}-p\right\| . \end{aligned}$$

It follows that

$$\begin{aligned} r\le \lim \inf _{n\rightarrow \infty }\left\| y_{n}-p\right\| \le \lim \sup _{n\rightarrow \infty }\left\| y_{n}-p\right\| \le r. \end{aligned}$$

This implies that

28 $$\begin{aligned} \lim _{n\rightarrow \infty }\left\| y_{n}-p\right\| =r. \end{aligned}$$

Since $\lim _{n\rightarrow \infty }\left\| x_{n}-p\right\| =r$ and (16), I see that

29 $$\begin{aligned} \lim \sup _{n\rightarrow \infty }\left\| S_{i\left( n\right) }^{k\left( n\right) }x_{n}-p\right\|&\le  \lim \sup _{n\rightarrow \infty }\left[ \begin{array}{c} \left\| x_{n}-p\right\| \\ +\mu _{n}\phi \left( \left\| x_{n}-p\right\| \right) +l_{n} \end{array} \right] \nonumber \\ & \le r. \end{aligned}$$

Notice that

$$\begin{aligned} \left\| S_{i\left( n\right) }^{k\left( n\right) }x_{n}-p+\delta _{n}\left( v_{n}-x_{n}\right) \right\| \le \left\| S_{i\left( n\right) }^{k\left( n\right) }x_{n}-p\right\| +\delta _{n}\left\| v_{n}-x_{n}\right\| . \end{aligned}$$

It follows that

30 $$\begin{aligned} \lim \sup _{n\rightarrow \infty }\left\| S_{i\left( n\right) }^{k\left( n\right) }x_{n}-p+\delta _{n}\left( v_{n}-x_{n}\right) \right\| \le r, \end{aligned}$$

and

31 $$\begin{aligned} \left\| x_{n}-p+\delta _{n}\left( v_{n}-x_{n}\right) \right\| \le \left\| x_{n}-p\right\| +\delta _{n}\left\| v_{n}-x_{n}\right\| , \end{aligned}$$

which implies that

32 $$\begin{aligned} \lim \sup _{n\rightarrow \infty }\left\| x_{n}-p+\delta _{n}\left( v_{n}-x_{n}\right) \right\| \le r, \end{aligned}$$

and I have that

33 $$\begin{aligned} y_{n}-p&=  \beta _{n}\left( S_{i\left( n\right) }^{k\left( n\right) }x_{n}-p+\delta _{n}\left( v_{n}-x_{n}\right) \right) +\left( 1-\beta _{n}\right) \left( x_{n}-p+\delta _{n}\left( v_{n}-x_{n}\right) \right) . \end{aligned}$$

Hence,

34 $$\begin{aligned} r= & {} \lim _{n\rightarrow \infty }\left\| y_{n}-p\right\| \nonumber \\= & {} \lim _{n\rightarrow \infty }\left\| \begin{array}{c} \beta _{n}\left( S_{i\left( n\right) }^{k\left( n\right) }x_{n}-p+\delta _{n}\left( v_{n}-x_{n}\right) \right) \\ +\left( 1-\beta _{n}\right) \left( x_{n}-p+\delta _{n}\left( v_{n}-x_{n}\right) \right) \end{array} \right\| . \end{aligned}$$

Using (30), (32), (34) and Lemma 2, I find

35 $$\begin{aligned} \lim _{n\rightarrow \infty }\left\| x_{n}-S_{i\left( n\right) }^{k\left( n\right) }x_{n}\right\| =0. \end{aligned}$$

Notice that

$$\begin{aligned} \left\| T_{i\left( n\right) }^{k\left( n\right) }x_{n}-x_{n}\right\|&\le \left\| T_{i\left( n\right) }^{k\left( n\right) }x_{n}-y_{n}\right\| +\left\| T_{i\left( n\right) }^{k\left( n\right) }y_{n}-x_{n}\right\| \\ &\le {} \left\| x_{n}-y_{n}\right\| +\mu _{n}\phi \left( \left\| x_{n}-y_{n}\right\| \right) +l_{n} +\left\| T_{i\left( n\right) }^{k\left( n\right) }y_{n}-x_{n}\right\| \\ &\le \beta _{n}\left\| x_{n}-S_{i\left( n\right) }^{k\left( n\right) }x_{n}\right\| +\delta _{n}\left\| v_{n}-x_{n}\right\| \\ &\quad +\mu _{n}\phi \left( \left\| x_{n}-y_{n}\right\| \right) +l_{n}+\left\| T_{i\left( n\right) }^{k\left( n\right) }y_{n}-x_{n}\right\| . \end{aligned}$$

It follows from (16), (27) and (35) that

36 $$\begin{aligned} \lim _{n\rightarrow \infty }\left\| x_{n}-T_{i\left( n\right) }^{k\left( n\right) }x_{n}\right\| =0. \end{aligned}$$

Notice that

$$\begin{aligned} \left\| y_{n}-x_{n}\right\| &= \left\| \beta _{n}\left( S_{i\left( n\right) }^{k\left( n\right) }x_{n}-x_{n}\right) +\delta _{n}\left( v_{n}-x_{n}\right) \right\| \\ &\le  \beta _{n}\left\| S_{i\left( n\right) }^{k\left( n\right) }x_{n}-x_{n}\right\| +\delta _{n}\left\| v_{n}-x_{n}\right\| . \end{aligned}$$

In view of (35), I see from the restriction (iii) that

37 $$\begin{aligned} \lim _{n\rightarrow \infty }\left\| y_{n}-x_{n}\right\| =0. \end{aligned}$$

Notice that

$$\begin{aligned} \left\| x_{n-1}-T_{i\left( n\right) }^{k\left( n\right) }x_{n}\right\| &\le \left\| x_{n-1}-T_{i\left( n\right) }^{k\left( n\right) }y_{n}\right\| +\left\| T_{i\left( n\right) }^{k\left( n\right) }y_{n}-T_{i\left( n\right) }^{k\left( n\right) }x_{n}\right\| \\ &\le  \left\| x_{n-1}-T_{i\left( n\right) }^{k\left( n\right) }y_{n}\right\| +\left\| y_{n}-x_{n}\right\| +\mu _{n}\phi \left( \left\| y_{n}-x_{n}\right\| \right) +l_{n}. \end{aligned}$$

It follows that (16), (26) and (37) that

38 $$\begin{aligned} \lim _{n\rightarrow \infty }\left\| x_{n-1}-T_{i\left( n\right) }^{k\left( n\right) }x_{n}\right\| =0. \end{aligned}$$

Notice that

$$\begin{aligned} \left\| x_{n}-x_{n-1}\right\| \le \alpha _{n}\left\| T_{i\left( n\right) }^{k\left( n\right) }y_{n}-x_{n-1}\right\| +\gamma _{n}\left\| u_{n}-x_{n-1}\right\| . \end{aligned}$$

In view of (26), I see from the restriction (iii) that

39 $$\begin{aligned} \lim _{n\rightarrow \infty }\left\| x_{n}-x_{n-1}\right\| =0. \end{aligned}$$

Notice that

$$\begin{aligned} \left\| T_{i\left( n\right) }^{k\left( n\right) }x_{n}-S_{i\left( n\right) }^{k\left( n\right) }x_{n}\right\| \le \left\| T_{i\left( n\right) }^{k\left( n\right) }x_{n}-x_{n}\right\| +\left\| x_{n}-S_{i\left( n\right) }^{k\left( n\right) }x_{n}\right\| . \end{aligned}$$

It follows from (35) and (36) that

$$\begin{aligned} \lim _{n\rightarrow \infty }\left\| T_{i\left( n\right) }^{k\left( n\right) }x_{n}-S_{i\left( n\right) }^{k\left( n\right) }x_{n}\right\| =0. \end{aligned}$$

From (39), I also have

40 $$\begin{aligned} \lim _{n\rightarrow \infty }\left\| x_{n}-x_{n+j}\right\| =0,\quad \forall j=1,2,\ldots ,N. \end{aligned}$$

For any positive $n>N$, it can be rewritten as $n=\left( k\left( n\right) -1\right) N+i\left( n\right)$, $i\left( n\right) \in \left\{ 1,2,\ldots ,N\right\}$. Note that

41 $$\begin{aligned} \left\| x_{n-1}-T_{n}x_{n}\right\| &\le \left\| x_{n-1}-T_{i\left( n\right) }^{k\left( n\right) }x_{n}\right\| +\left\| T_{i\left( n\right) }^{k\left( n\right) }x_{n}-T_{n}x_{n}\right\| \nonumber \\ &\le \left\| x_{n-1}-T_{i\left( n\right) }^{k\left( n\right) }x_{n}\right\| +L\left\| T_{i\left( n\right) }^{k\left( n\right) -1}x_{n}-x_{n}\right\| \nonumber \\ &\le \left\| x_{n-1}-T_{i\left( n\right) }^{k\left( n\right) }x_{n}\right\| +L\left\{ \left\| T_{i\left( n\right) }^{k\left( n\right) -1}x_{n}-T_{i\left( n-N\right) }^{k\left( n\right) -1}x_{n-N}\right\| \right. \nonumber \\ &\left. \quad +\left\| T_{i\left( n-N\right) }^{k\left( n\right) -1}x_{n-N}-x_{\left( n-N\right) -1}\right\| +\left\| x_{\left( n-N\right) -1}-x_{n}\right\| \right\} . \end{aligned}$$

On the other hand, I have $n-N=\left( \left( k\left( n\right) -1\right) -1\right) N+i\left( n\right) =\left( \left( k\left( n\right) -1\right) -1\right) N+i\left( n-N\right) ,$ i.e.,

$$\begin{aligned} k\left( n-N\right) =k\left( n\right) -1\text { and }i\left( n-N\right) =i\left( n\right) . \end{aligned}$$

It follows that

42 $$\begin{aligned} \left\| T_{i\left( n\right) }^{k\left( n\right) -1}x_{n}-T_{i\left( n-N\right) }^{k\left( n\right) -1}x_{n-N}\right\| \le L\left\| x_{n}-x_{n-N}\right\| , \end{aligned}$$

and

43 $$\begin{aligned} \left\| T_{i\left( n-N\right) }^{k\left( n\right) -1}x_{n-N}-x_{\left( n-N\right) -1}\right\| =\left\| T_{i\left( n-N\right) }^{k\left( n-N\right) }x_{n-N}-x_{\left( n-N\right) -1}\right\| . \end{aligned}$$

Combining (38), (40) with (41), I see that

44 $$\begin{aligned} \lim _{n\rightarrow \infty }\left\| x_{n-1}-T_{n}x_{n}\right\| =0. \end{aligned}$$

On the other hand, I have that

$$\begin{aligned} \left\| x_{n}-T_{n}x_{n}\right\| \le \left\| x_{n}-x_{n-1}\right\| +\left\| x_{n-1}-T_{n}x_{n}\right\| . \end{aligned}$$

From (39) and (44), I obtain that

45 $$\begin{aligned} \lim _{n\rightarrow \infty }\left\| x_{n}-T_{n}x_{n}\right\| =0. \end{aligned}$$

Consequently, for any $j=1,2,\ldots ,N$, I see that

$$\begin{aligned} \left\| x_{n}-T_{n+j}x_{n}\right\| &\le \left\| x_{n}-x_{n+j}\right\| +\left\| x_{n+j}-T_{n+j}x_{n+j}\right\| \\ &\quad +\left\| T_{n+j}x_{n+j}-T_{n+j}x_{n}\right\| \\ &\le \left( 1+L\right) \left\| x_{n}-x_{n+j}\right\| +\left\| x_{n+j}-T_{n+j}x_{n+j}\right\| . \end{aligned}$$

From (40) and (45), I arrive at

$$\begin{aligned} \lim _{n\rightarrow \infty }\left\| x_{n}-T_{n+j}x_{n}\right\| =0. \end{aligned}$$

Therefore, for $\forall i\in \left\{ 1,2,\ldots ,N\right\}$, there exists some $e\in \left\{ 1,2,\ldots ,N\right\}$ such that $n+e=i\left( \hbox {mod} N\right)$. It follows that

46 $$\begin{aligned} \lim _{n\rightarrow \infty }\left\| x_{n}-T_{i}x_{n}\right\| =\lim _{n\rightarrow \infty }\left\| x_{n}-T_{n+e}x_{n}\right\| =0. \end{aligned}$$

Similarly, by using the same argument as in the proof above, I have

47 $$\begin{aligned} \lim _{n\rightarrow \infty }\left\| x_{n}-S_{i}x_{n}\right\| =\lim _{n\rightarrow \infty }\left\| x_{n}-S_{n+e}x_{n}\right\| =0. \end{aligned}$$

Since

$$\begin{aligned} \left\| S_{i}x_{n}-T_{i}x_{n}\right\| \le \left\| S_{i}x_{n}-x_{n}\right\| +\left\| T_{i}x_{n}-x_{n}\right\| , \end{aligned}$$

I find from (46) and (47) that

48 $$\begin{aligned} \lim _{n\rightarrow \infty }\left\| S_{i}x_{n}-T_{i}x_{n}\right\| =0, \text { \ \ }\forall i\in \left\{ 1,2,\ldots ,N\right\} . \end{aligned}$$

This completes the proof. $\square$

### **Theorem 2**

*LetEbe a real uniformly convex Banach space*, *Kbe a nonempty,closed and convex subset ofE*. *Let*$\left\{ S_{i}\right\} _{i=1}^{N}$, $\left\{ T_{i}\right\} _{i=1}^{N}:K\rightarrow K$*be* 2*Nuniformly*$L_{i}\hbox{-}Lipschitz$*total asymptotically nonexpansive mappings*$\left\{ \mu _{n}\right\}$, $\left\{ l_{n}\right\}$*defined by* (1) *and* (2) *such that*

49 $$\begin{aligned} \sum \limits _{n=1}^{\infty }\mu _{n}<\infty ,\quad \sum \limits _{n=1}^{\infty }l_{n}<\infty \end{aligned}$$

*and*${\mathcal {F}}:\bigcap \nolimits _{i=1}^{N}F\left( T_{\dot{I}}\right) \cap F\left( S_{\dot{I}}\right) \ne \varnothing$. *Assume that there exist at least a*$T_{i} \left( i\in I\right)$*which is*$semi-compact$. *Assume that there existM*, $M^{*}>0$*such that*$\phi (\lambda )\le M^{*}\lambda$*for all*$\lambda \ge M$, $i\in \left\{ i=1,2,\ldots ,N\right\} .$*Let*$\left\{ u_{n}\right\}$*and*$\left\{ v_{n}\right\}$*are two bounded sequences inK*. *Let*$\left\{ \alpha _{n}\right\} ,\left\{ \beta _{n}\right\} ,\left\{ \gamma _{n}\right\}$*and*$\left\{ \delta _{n}\right\}$*be four real sequences in*$\left[ \frac{L-1}{L},a\right]$, *where*$L=\max _{1\le i\le N}\left\{ L_{i}\right\} >1$*andais some constant in*$\left( 0,1\right)$*satisfying the following conditions:*

(i) $\alpha _{n}+\gamma _{n}\le 1$*and*$\beta _{n}+\delta _{n}\le 1$*for all*$n\ge 1;$
(ii) $$\lim \sup _{n\rightarrow \infty }\alpha _{n}<1;$$
(iii) $$\sum \nolimits _{n=1}^{\infty }\gamma _{n}<\infty $$, $$\sum \nolimits _{n=1}^{ \infty }\delta _{n}<\infty .$$

*Then the sequence*$\left\{ x_{n}\right\}$*be defined by* (3) *converges strongly to a common fixed point of*$\left\{ T_{1},T_{2},\ldots ,T_{N},S_{1},S_{2},\ldots ,S_{N}\right\}$*inE*.

### *Proof*

Without loss of generality, I may assume that $T_{1}$ is semicompact. It follows from (46) that

50 $$\begin{aligned} \lim _{n\rightarrow \infty }\left\| x_{n}-T_{1}x_{n}\right\| =0. \end{aligned}$$

By the semicompactness of $T_{1}$, I have there exists a subsequence $\left\{ x_{n_{i}}\right\}$ of $\left\{ x_{n}\right\}$ such that $x_{n_{i}}\rightarrow q\in K$ strongly. From (46), I have

$$\begin{aligned} \lim _{n\rightarrow \infty }\left\| x_{n_{i}}-T_{l}x_{n_{i}}\right\| =\left\| q-T_{l}q\right\| =0, \end{aligned}$$

for all $l=1,2,\ldots ,N$. Also, it follows from (48) and (50), I have

$$\begin{aligned} \lim _{n\rightarrow \infty }\left\| x_{n_{i}}-S_{l}x_{n_{i}}\right\| =\left\| q-S_{l}q\right\| =0, \end{aligned}$$

for any $l\in I$. This implies that $q\in {\mathcal {F}}$. From Lemma 3 , I know that $\lim _{n\rightarrow \infty }\left\| x_{n}-q\right\|$ exists for all $q\in {\mathcal {F}}$. It follows from $x_{n_{i}}\rightarrow q$ that $\lim _{n\rightarrow \infty }\left\| x_{n}-q\right\| =0$. Thus, the iterative sequence $\left\{ x_{n}\right\}$ defined by (3) converges strongly to a common fixed point of $\left\{ T_{1},T_{2},\ldots ,T_{N},S_{1},S_{2},\ldots ,S_{N}\right\}$ in *E*. This completes the proof. $\square$

### **Corollary 1**

*LetEbe a real uniformly convex Banach space*, *Kbe a nonempty,closed and convex subset ofE*. *Let*$\left\{ T_{i}\right\} _{i=1}^{N}:K\rightarrow K$*beNuniformly*$L_{i}-Lipschitz$*total asymptotically nonexpansive mappings*$\left\{ \mu _{n}\right\}$, $\left\{ l_{n}\right\}$*defined by* (1) *such that*

51 $$\begin{aligned} \sum \limits _{n=1}^{\infty }\mu _{n}<\infty ,\sum \limits _{n=1}^{\infty }l_{n}<\infty \end{aligned}$$

*and*${\mathcal {F}}:\bigcap \nolimits _{i=1}^{N}F\left( T_{\dot{I}}\right) \ne \varnothing$. *Assume that there existM*, $M^{*}>0$*such that*$\phi (\lambda )\le M^{*}\lambda$*for all*$\lambda \ge M$, $i\in \left\{ i=1,2,\ldots ,N\right\} .$*Let*$\left\{ x_{n}\right\}$*be the sequence defined by*

$$\begin{aligned} \left\{ \begin{array}{l} n=\left( k\left( n\right) -1\right) +i\left( n\right) \in \left\{ 1,\ldots ,N\right\} , \\ x_{n}=\left( 1-\alpha _{n}-\gamma _{n}\right) x_{n-1}+\alpha _{n}T_{i\left( n\right) }^{k\left( n\right) }y_{n}+\gamma _{n}u_{n}, \\ y_{n}=\left( 1-\beta _{n}-\delta _{n}\right) x_{n}+\beta _{n}T_{i\left( n\right) }^{k\left( n\right) }x_{n}+\delta _{n}v_{n},\text { }n\ge 1, \end{array} \right. \end{aligned}$$

*where*$\left\{ u_{n}\right\}$*and*$\left\{ v_{n}\right\}$*are two bounded sequences inK*. *Let*$\left\{ \alpha _{n}\right\} ,\left\{ \beta _{n}\right\} ,\left\{ \gamma _{n}\right\}$*and*$\left\{ \delta _{n}\right\}$*be four real sequences in*$\left[ \frac{L-1}{L},a\right]$, *where*$L=\max _{1\le i\le N}\left\{ L_{i}\right\} >1$*andais some constant in*$\left( 0,1\right)$*satisfying the following conditions:*

(i) $\alpha _{n}+\gamma _{n}\le 1$*and*$\beta _{n}+\delta _{n}\le 1$*for all*$n\ge 1;$
(ii) $$\lim \sup _{n\rightarrow \infty }\alpha _{n}<1;$$
(iii) $$\sum \nolimits _{n=1}^{\infty }\gamma _{n}<\infty $$, $$\sum \nolimits _{n=1}^{ \infty }\delta _{n}<\infty .$$

*Then*

- *The implicit iterative sequence*$\left\{ x_{n}\right\}$*converges strongly to a common fixed point in*${\mathcal {F}}$*if and only if*$\lim \inf _{n\rightarrow \infty }d\left( x_{n},{\mathcal {F}}\right) =0$, *where*$d\left( x,{\mathcal {F}}\right)$*denotes the distance ofxto set*${\mathcal {F}}$, *i.e*., $d\left( x,{\mathcal {F}}\right) =\inf _{y\in {\mathcal {F}}}d\left( x,y\right) .$
- *If one of the mappings in*$$\left\{ T_{1},\ldots ,T_{N}\right\} $$ is $$ semi-compact$$, *then the sequence*$$\left\{ x_{n}\right\} $$*converges strongly to a common fixed point of*$$\left\{ T_{1},T_{2},\ldots ,T_{N}\right\} .$$

### **Corollary 2**

*LetEbe a real uniformly convex Banach space*, *Kbe a nonempty,closed and convex subset ofE*. *Let*$\left\{ T_{i}\right\} _{i=1}^{N}:K\rightarrow K$*beNuniformly*$L_{i}-Lipschitz$*total asymptotically nonexpansive mappings*$\left\{ \mu _{n}\right\}$, $\left\{ l_{n}\right\}$*defined by* (1) *such that*

52 $$\begin{aligned} \sum \limits _{n=1}^{\infty }\mu _{n}<\infty ,\quad \sum \limits _{n=1}^{\infty }l_{n}<\infty \end{aligned}$$

*and*${\mathcal {F}}:\bigcap \nolimits _{i=1}^{N}F\left( T_{\dot{I}}\right) \ne \varnothing$. *Assume that there existM*, $M^{*}>0$*such that*$\phi (\lambda )\le M^{*}\lambda$*for all*$\lambda \ge M$, $i\in \left\{ i=1,2,\ldots ,N\right\} .$*Let*$\left\{ x_{n}\right\}$*be the sequence defined by*

$$\begin{aligned} \left\{ \begin{array}{l} n=\left( k\left( n\right) -1\right) +i\left( n\right) \in \left\{ 1,\ldots ,N\right\} , \\ x_{n}=\left( 1-\alpha _{n}-\gamma _{n}\right) x_{n-1}+\alpha _{n}T_{i\left( n\right) }^{k\left( n\right) }x_{n}+\gamma _{n}u_{n},\text { }n\ge 1, \end{array} \right. \end{aligned}$$

*where*$\left\{ u_{n}\right\}$*is a bounded sequence inK*. *Let*$\left\{ \alpha _{n}\right\}$*and*$\left\{ \gamma _{n}\right\}$*be two real sequences in*$\left[ \frac{L-1}{L},a\right]$, *where*$L=\max _{1\le i\le N}\left\{ L_{i}\right\} >1$*andais some constant in*$\left( 0,1\right)$*satisfying the following conditions:*

(i) $\alpha _{n}+\gamma _{n}\le 1$*and*$\beta _{n}+\delta _{n}\le 1$*for all*$n\ge 1;$
(ii) $$0<\lim \inf _{n\rightarrow \infty }\alpha _{n}<\lim \sup _{n\rightarrow \infty }\alpha _{n}<1;$$
(iii) $$\sum \nolimits _{n=1}^{\infty }\gamma _{n}<\infty $$.

*Then*

- *The implicit iterative sequence*$\left\{ x_{n}\right\}$*converges strongly to a common fixed point in*${\mathcal {F}}$*if and only if*$\lim \inf _{n\rightarrow \infty }d\left( x_{n},{\mathcal {F}}\right) =0$, *where*$d\left( x,{\mathcal {F}}\right)$*denotes the distance ofxto set*${\mathcal {F}}$, *i.e*., $d\left( x,{\mathcal {F}}\right) =\inf _{y\in {\mathcal {F}}}d\left( x,y\right) .$
- *If one of the mappings in*$$\left\{ T_{1},\ldots ,T_{N}\right\} $$ is $$ semi-compact$$, *then the sequence*$$\left\{ x_{n}\right\} $$*converges strongly to a common fixed point of*$$\left\{ T_{1},T_{2},\ldots ,T_{N}\right\} .$$

### *Remark 1*

Since total asymptotically nonexpansive mappings include asymptotically nonexpansive mappings, Theorem 2 improves and generalizes Theorem 3.7 in Cianciaruso et al. (2010) and Theorem 3.7 in Hao et al. (2012).

### **Conclusion 1**

1. *My theorems and corolaries which include the corresponding results announced in* Xu and Ori (2001), Sun (2003), Chang et al. (2006), Gu (2006) *as special cases fundamentally improve and generalize the results of* Cianciaruso et al. (2010) *and* Hao et al. (2012) *in the following sense*.
  (i) *Extend the mappings from the class of asimptotically nonexpansive mappings to the class of total asimptotically nonexpansive mappings*.
  (ii) *Extend the mappings from*$\left\{ T_{i}\right\} _{i=1}^{N}:K\rightarrow K$*beNmappings to*$\left\{ S_{i}\right\} _{i=1}^{N}$, $\left\{ T_{i}\right\} _{i=1}^{N}:K\rightarrow K$ be 2*Nmappings*.
2. *I considered an Ishikawa-type iterative algorithm for the class of total asimptotically nonexpansive mappings. But, my results are also available in the Mann-type iterative algorithm for the class of total asimptotically nonexpansive mappings*.

### *Example 1*

Let *E* is the real line with the usual norm $\left| .\right|$, $K=\left( -1,1\right)$. Assume that $Tx=\sin x$ and $Sx=\sin \left( -x\right)$ for $x\in K$. Let $\phi$ be a strictly increasing continuous function such that $\phi : \mathbb {R} ^{+}\rightarrow \mathbb {R}^{+}$ with $\phi (0)=0$. Let $\{\mu _{n}\}_{n\ge 1}$ and $\{l_{n}\}_{n\ge 1}$ in $\mathbb {R}$ be two sequences defined by $\mu _{n}=\frac{1}{n}$ and $l_{n}=\frac{1}{n+1}$, for all $n\ge 1$ ($\lim _{n\rightarrow \infty }\mu _{n}=\lim _{n\rightarrow \infty }\frac{1}{n}=0$, $\lim _{n\rightarrow \infty }l_{n}=\frac{1}{n+1}=0$). Since $Tx=\sin x$ for $x\in K$, I have

$$\begin{aligned} \left| T^{n}x-T^{n}y\right| \le \left| x-y\right| . \end{aligned}$$

For all *x*, $y\in K,$ I obtain

$$\begin{aligned}&\left| T^{n}x-T^{n}y\right| -\left| x-y\right| -\mu _{n}\phi (\left| x-y\right| )-l_{n} \\&\quad \le \left| x-y\right| -\left| x-y\right| -\mu _{n}\phi (\left| x-y\right| )-l_{n} \\&\quad \le 0 \end{aligned}$$

for all $n=1,2,\ldots$, $\{\mu _{n}\}_{n\ge 1}$ and $\{l_{n}\}_{n\ge 1}$ with $\mu _{n}$, $l_{n}\rightarrow 0$ as $n\rightarrow \infty$ and so *T* is a total asymptoticaly nonexpansive mapping. Also, $Sx=\sin \left( -x\right)$ for $x\in K$, I have

$$\begin{aligned} \left| S^{n}x-S^{n}y\right| \le \left| x-y\right| . \end{aligned}$$

For all *x*, $y\in K,$ I obtain

$$\begin{aligned}&\left| S^{n}x-S^{n}y\right| -\left| x-y\right| -\mu _{n}\phi (\left| x-y\right| )-l_{n} \\&\quad \le \left| x-y\right| -\left| x-y\right| -\mu _{n}\phi (\left| x-y\right| )-l_{n} \\&\quad \le 0 \end{aligned}$$

for all $n=1,2,\ldots$, $\{\mu _{n}\}_{n\ge 1}$ and $\{l_{n}\}_{n\ge 1}$ with $\mu _{n}$, $l_{n}\rightarrow 0$ as $n\rightarrow \infty$ and so *S* is a total asymptoticaly nonexpansive mapping. Clearly, $F:=F\left( T\right) \cap F\left( S\right) =\left\{ 0\right\}$. Set

$$\begin{aligned} \alpha _{n}=\beta _{n}=\frac{n}{2n+1},\text { }\delta _{n}=\gamma _{n}=\frac{ n^{3}}{6n^{3}+1}\text { and }u_{n}=v_{n}=\frac{1}{n+1} \end{aligned}$$

for $n\ge 1$. In order to easily calculate, I modifed my iteration scheme for $n=1$. This scheme (53) is defined as follows:

53 $$\begin{aligned} x_{1} &= \left( 1-\alpha _{1}-\gamma _{1}\right) x_{0}+\alpha _{1}T_{i\left( 1\right) }^{k\left( 1\right) }y_{1}+\gamma _{1}u_{1}, \nonumber \\ y_{1}&=  {} \left( 1-\beta _{1}-\delta _{1}\right) x_{1}+\beta _{1}S_{i\left( 1\right) }^{k\left( 1\right) }x_{1}+\delta _{1}v_{1}. \end{aligned}$$

The numerical experiment outcome obtained by using Scientific WorkPlace 5.5 show that as $x_{0}=0$, the computation of $y_{1}=9.0853\times 10^{-2}$. This example illustrates the efficiency of approximation of common fixed points of total asymptotically nonexpansive mappings.

Let $\left\{ S_{i}\right\} _{i=1}^{N}$, $\left\{ T_{i}\right\} _{i=1}^{N}:E\rightarrow E$ be 2*N* total asymptotically nonexpansive mappings; assuming existence of common fixed points of these operators, our theorems and method of proof easily carry over to this class of mappings using the following implicit iterative scheme $\left\{ x_{n}\right\}$ with errors:

54 $$\begin{aligned} \left\{ \begin{array}{l} x_{0}\in K, \\ x_{n}=a_{n}x_{n-1}+b_{n}T_{i\left( n\right) }^{k\left( n\right) }y_{n}+c_{n}u_{n}, \\ y_{n}=a_{n}^{^{\prime }}x_{n}+b_{n}^{^{\prime }}S_{i\left( n\right) }^{k\left( n\right) }x_{n}+c_{n}^{^{\prime }}v_{n},\text { }n\ge 1 \end{array} \right. \end{aligned}$$

where $n=\left( k\left( n\right) -1\right) N+i\left( n\right)$, $i\left( n\right) \in 1,2,\ldots ,N$, $\left\{ a_{n}\right\}$, $\left\{ b_{n}\right\}$, $\left\{ c_{n}\right\}$, $\left\{ a_{n}^{^{\prime }}\right\}$, $\left\{ b_{n}^{^{\prime }}\right\}$, $\left\{ c_{n}^{^{\prime }}\right\}$ are six real sequences in $\left[ 0,1\right]$ satisfying $a_{n}= b_{n}=c_{n}=1=a_{n}^{^{\prime }}= b_{n}^{^{\prime }}=c_{n}^{^{\prime }}$ for all $n\ge 1$, $\left\{ u_{n}\right\}$ and $\left\{ v_{n}\right\}$ are two bounded sequences.

In order not to enlarge this note unnecessarily, I only include total asymptotically nonexpansive mappings. But, in accordance with the above proof of theorem, one can easily prove in total asymptotically quasi nonexpansive mappings.

If $S=I$, then (54) transform to implicit iterative scheme defined by Mukhamedov and Saburov (see, more details Mukhamedov and Saburov 2012a). My theorems and corolaries also improve and generalize the mappings from the class of a finite family of quasi-asimptotically nonexpansive mappings to the class of a finite family of total quasi-asimptotically nonexpansive mappings.

If *S*, $T:E\rightarrow E$ be two total asymptotically nonexpansive mappings and take $S=I$, then (54) reduce to implicit iterative scheme defined by Mukhamedov and Saburov (see, more details Mukhamedov and Saburov 2011). My theorems and corolaries also improve and generalize the mappings from the class of quasi-asimptotically nonexpansive mappings to the class of total quasi-asimptotically nonexpansive mappings.
